# Efficacy of *Acacia nilotica* Linn. Pod's Sitz Bath plus Vaginal Pessary in Syndromic Management of Abnormal Vaginal Discharge: A Randomized Controlled Trial

**DOI:** 10.1155/2022/5769555

**Published:** 2022-05-25

**Authors:** Rushda Saeedi, Arshiya Sultana, Khaleequr Rahman, Md Belal Bin Heyat, Mohammad Amjad Kamal, Mumuni Ishawu

**Affiliations:** ^1^Department of Amraze Niswan wa Ilmul Qabalat, National Institute of Unani Medicine, Ministry of AYUSH, Government of India, Bengaluru 560091, Karnataka, India; ^2^Department of Ilmul Saidla, National Institute of Unani Medicine, Ministry of AYUSH, Government of India, Bengaluru 560091, Karnataka, India; ^3^IoT Research Center, College of Computer Science and Software Engineering, Shenzhen University, Shenzhen, Guangdong 518060, China; ^4^International Institute of Information Technology, Hyderabad, Telangana 500032, India; ^5^Department of Science and Engineering, Novel Global Community Educational Foundation, Hebersham, NSW 2770, Australia; ^6^Institute for Systems Genetics, Frontier Science Center for Disease-related Molecular Network, West China Hospital, Sichuan University, Chengdu 610041, Sichuan, China; ^7^King Fahd Medical Research Center, King Abdulaziz University, Saudi Arabia; ^8^Department of Pharmacy, Faculty of Allied Health Sciences, Daffodil International University, Bangladesh; ^9^Enzymoics, Novel Global Community Educational Foundation, Hebersham, NSW 2770, Australia; ^10^Faculty of Business and Management Studies, Koforidua Technical University, Koforidua, Ghana

## Abstract

**Objectives:**

Abnormal vaginal discharge (*Sayalan al-Rahim*) is a common public health problem that significantly disrupts the health-related quality of life (HRQoL). Syndromic management infers the concurrent treatment of two or more infections. Hence, a comparative, single-blind study was planned to determine the efficacy of *Acacia* (*Acacia nilotica* Linn.) pod's sitz bath (*Abzan*) plus vaginal pessary (*Farzaja*) vs. placebo in abnormal vaginal discharge syndromic management, its associated symptoms, and women's HRQoL.

**Methods:**

Diagnosed patients (*n* = 66) were randomly divided into *Acacia* (*n* = 33) and placebo (*n* = 33) group. *Acacia* group received Sitz bath with *Acacia* pod powder (30g) solution followed by vaginal cotton pessary (5 ml of the same solution) once daily for 10 days. The placebo group received palm sugar powder (30g) solution for Sitz bath plus vaginal cotton pessary same as the *Acacia* group. Primary outcomes included clinical cure assessed with VAS for symptoms and Modified McCormack Pain Scale (McPS) for pelvic tenderness. The secondary outcomes included were the EQ-5D-5 L questionnaire, TSQM questionnaire, sachet count, and microbiological cure. Overall, therapeutic cure included clinical and microbiological cure after treatment.

**Results:**

The overall therapeutic cure for bacterial vaginosis, cervicitis, and uncomplicated pelvic inflammatory disease was 100% (*n* = 7/7), 45.45% (*n* = 10/22), and 71.42% (*n* = 5/7), respectively, in the *Acacia* group, while in the placebo group none of the patients had responded. The VAS score for symptoms was significantly reduced in *Acacia* than in the placebo group. At each follow-up, the improvement in the EQ-5D-5 L level of HRQoL was significantly higher in the *Acacia* group than in the placebo group.

**Conclusion:**

*Acacia* would be an effective and safe alternative in syndromic management of abnormal vaginal discharge, associated symptoms, and improved women's HRQoL. *Trial registration*. This trial was registered in the Clinical Trials Registry of Indian Trials Website and given the identification no. CTRI/2018/02/012175 (dated: 27/02/2018).

## 1. Introduction

Abnormal vaginal discharge (*Sayalan al-Rahim*) is the commonest gynaecological problem in women [1] reported (21.8%) in reproductive tract infection (RTI) after abnormal uterine bleeding (AUB). Almost ten million clinical visits each year are ascribed to vaginal discharge complaints. It is more common in women in developing countries, and available evidence suggests that about one-fourth of these women are having this complaint [2]. The ICMR conducted a hospital-based study in Delhi and reported the prevalence of abnormal vaginal discharge (AVD) is about 30% in women with RTIs. *Gardnerella vaginalis*, *Trichomonas vaginalis*, *Candida*, aerobic and anaerobic microbes, viruses, *Chlamydia*, etc. are various organisms that cause RTIs [1]. Lower reproductive tract infections are more common in Indian reproductive age women [3]. These infections can lead to long-term complications in women such as infertility, ectopic pregnancy, pelvic inflammatory disease, and propensity towards neoplasia with noteworthy morbidity that affects the quality of life and leads to a substantial burden on the healthcare system [4]. Abnormal white/vaginal discharge in women is associated with various other symptoms such as pruritus vulvae, backache, dysuria, and burning micturition. The syndromic approach (WHO 2005) is employed to manage vaginal infections by healthcare providers. Syndromic management is established on the patient's symptoms and infers the concurrent treatment of two or more infections [5]. Clinically, depending on the pathogenic infection, the first choice to treat infective vaginal discharge is antibiotics. To cure or prevent infections, no single broad-spectrum formulations are currently available for intravaginal use. Most of the drugs used to treat infective vaginal discharge cause side effects. Furthermore, antimicrobial resistance is increasing, rendering some regimens ineffective in several infections [3]. In the current scenario, adverse drug reactions of a chemical drug are one of the reasons for an increased drive observed towards the consumption of herbal drugs in many disease conditions. One of the suggested herbal drugs is *Acacia* (*Acacia nilotica* Linn.) pod for abnormal vaginal discharge.

*A. arabica* is a moderate-sized, spiny evergreen tree. It is a popular ornamental multipurpose avenue tree from the family Fabaceae and is commonly known as *kikar*. In India, its parts are used in ethnomedicinal practice for the prevention and treatment of various illnesses for many years [6]. In classical Unani medical texts, it has been mentioned that *A. arabica* pods are useful in abnormal vaginal discharge as it possesses anti-inflammatory (*Muhallil al-Waram*), astringent (*Qabiz*), and antiseptic (*Daf'-i-Taffun*) properties [6,7]. Satish et al. (2008) demonstrated the activity of pod *A. arabica* against a few strains of bacteria and fungi [8]. Kalaivani and Methew (2010) also demonstrated the maximum activity of *A. arabica* against *Candida albicans* and anaerobic bacteria [9]. Pharmacologically, this medicinal plant is proven for astringent, antimicrobial, anti-inflammatory, analgesic, antioxidant, and diuretic properties [10–14]. Although a few studies have been carried out on complementary and alternative medicines for abnormal vaginal discharge, bacterial vaginosis and cervicitis are available [3,5,15–20]. However, to date, none of the studies has determined the efficacy of *Acacia* pod's Sitz bath and vaginal pessary in syndromic management of abnormal vaginal discharge, associated symptoms, and women's health-related quality of life (HRQoL). Hence, this study was planned to validate its efficacy.

## 2. Materials and Methods

### 2.1. Trial Design

A parallel, single-blind, prospective, single-centre, simple randomized, placebo-controlled trial was carried out with approval from Institutional Ethical Committee (IEC No.NIUM/IEC/2016-17/014/ANQ/06). The study was conducted based on the GCP guidelines, Ministry of AYUSH, Govt. of India, and the Helsinki Declaration. All randomized patients received written and verbal information about the aims and procedures of the research and then signed a consent form to participate in the study. Patients at any point in time had the option to withdraw from the study.

### 2.2. Participants

Sixty-six patients with abnormal vaginal discharge were recruited from the outpatients and inpatients of our hospital

#### 2.2.1. Inclusion and Exclusion Criteria

Married women between 18 and 50 years of age who had abnormal vaginal discharge and/or associated with low backache, burning micturition, lower abdomen pain, dysuria, dyspareunia, vulvar itching, and irritation were eligible for inclusion.

Patients were excluded from the study, who had undiagnosed uterine or vaginal bleeding, ulceration, vaginal douches, and genital malignancies. Pregnant, lactating, and unmarried women who were suspicious or clinically manifested with venereal disease were also excluded.

#### 2.2.2. Demographic and Clinical Assessment

A general questionnaire including demographic characteristics and relevant history was completed for each patient. The patient's socio-economic status was recorded as per Kuppuswamy's socio-economic scale. At visit 1 (Day 0), the VAS score was calculated for abnormal vaginal discharge and its associated symptoms. The per speculum and vaginum examination were performed to note the Modified McCormack tenderness scale for pelvic pain, the nature, colour, quantity, and consistency of vaginal discharge and other associated clinical features of infections (vaginitis, cervicitis, cervical ectopy, and uncomplicated pelvic inflammatory disease (uPID). A vaginal wet mount test to diagnose bacterial vaginosis, candidiasis and trichomoniasis, and uPID was performed. All laboratory procedures were performed in the pathological laboratory of the National Institute of Unani Medicine. BV was diagnosed with Amsel criteria. Cervicitis was diagnosed with the presence of congestion, hypertrophy, erosion of the cervix along with the presence of uterine/cervical motion tenderness, thick yellowish or greyish discharge, and the presence of >10 WBC per HPF on saline microscopy. Uncomplicated PID (uPID) was diagnosed as per CDC guidelines the presence of at least one of the following, i.e., uterine tenderness, cervical motion tenderness, or adnexal tenderness, and one or more of the following additional criteria, i.e., abnormal cervical or vaginal thick discharge; the presence of >10 WBCs per HPF or abundant number of WBCs with saline microscopy of vaginal fluid and elevated erythrocyte sedimentation rate were noted. The severity of cervical ectopy was graded as 2a (1/3rd) if it was involved 1/3rd portion of the cervix around the os and 2b (2/3rd) if the involved portion was 2/3rd of the cervix. Routine investigations at baseline were performed to exclude general diseases and sexually transmitted diseases. Wet mount and pap's smears were done at visit 1 (Day 0) and postintervention (days 11–13) for the assessment of the efficacy of the test drug. Pelvic ultrasonography was performed to exclude genital malignancies and other pathologies, respectively, at baseline.

### 2.3. Intervention

**Figure 1:**
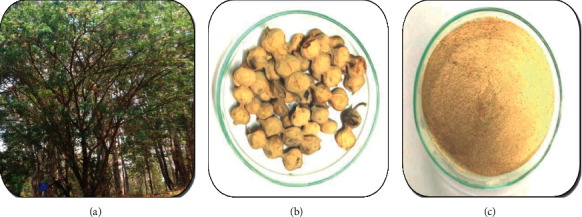
Depiction of test drug (*Acacia nilotica*): (a) tree, (b) pod, (c) powder of aqueous extract.

After a thorough literature survey, *Acacia* pod's Sitz bath plus vaginal cotton pessary for abnormal vaginal discharge was selected from the classical Unani literature based on its Unani and pharmacological properties such as antimicrobial, anti-inflammatory, and astringent. The pharmacognosist, Dr S. Noorunnisa Begum (Senior Assistant Professor, Centre for Repository of Medicinal Resources, Trans-Disciplinary University, Bengaluru), authenticated and identified the test drug as pods of *Acacia nilotica* Linn. belonging to the family Fabaceae with specimen number FRLHT Acc. No. 5008. The common name is *Acacia*. The test drug has been deposited in the Department of Pharmacology of our Institute with voucher specimen number 56/UQ/Res/2019 for future reference [Figure 1]

#### 2.3.1. Extract Preparation

*Joshanda* of pods was modified into dry powder. Dried pods of *A. arabica* Linn. were coarsely powdered and sieved under aseptic precautions. To obtain the extract, the sieved powder was soaked in water (at the ratio of 1 : 4) and boiled at 100°C for an hour and filtered. The filtrate was dried in a hot air oven for four hours at 60°C, and the dry powder was obtained. All procedures were carried out in our pharmacy under the direct supervision of the pharmacist from our research team.

#### 2.3.2. Dispensing of Drugs

To avoid exposure to humidity, 30g extract powder or placebo (palm sugar powder) was packed and dispensed in airtight aluminium sachets. Ten aluminium sachets were dispensed to each patient. One placebo capsule filled with edible cellulose (250 mg) was administered orally in the morning after meal for 10 days in both groups to increase the compliance of the patients.

#### 2.3.3. Dosage and Methods

Patients were advised for a Sitz bath with powder followed by per vaginum cotton pessary soaked in 5 ml of the same solution once daily for 10 days in both groups. Patients were taught verbally how to use their medication for sitz bath and vaginal pessary insertion. All patients were instructed to add and mix the powder of one sachet in 250 ml of lukewarm water, and from this, 5 ml of solution was kept aside for the vaginal pessary and the remaining solution was added in 5 litres of water in a sitz bath for 20 min. After the sitz bath, patients were instructed to insert a vaginal pessary per vagina soaked in 5 ml of solution (which was kept aside) and to remove it the next morning.

### 2.4. Follow-Up

To minimize the dropout rate, patients were instructed to visit the hospital on Day 3 and days 11–14 during treatment and two follow-ups on days 30–34 and Day 45 without treatment. If patients were not able to visit on Day 3, they were called on mobile to enquire regarding the clinical features and compliance of the research drug. Moreover, they were asked to deliver empty and unused sachets. The side effects were assessed by the researcher, who determined whether the event was study related or not.

### 2.5. Outcomes

The primary outcomes (clinical and symptomatic response) included a change in VAS score for symptoms (abnormal vaginal discharge, lower abdominal pain, dysuria, burning micturition, dyspareunia, vulvar irritation, and itching) and Modified McCormack Pain Scale for abdominal pain and rebound tenderness at Day 11 and Day 45 from baseline.

The secondary outcome included changes in quality of life assessed by the EQ-5D-5 L health survey questionnaire from baseline to days 11–14, days 30–34, and Day 45. Treatment Satisfaction Questionnaire for Medication (TSQM), Ver II for satisfaction with medications, sachets count for compliance were assessed on days 11–14 after completion of the treatment and microbiological cure (vaginal wet mount test, pH and Pap's smear) was assessed on days 11–14 from baseline.

### 2.6. Sachet's Count

All patients were given ten sachets at baseline. Patients were instructed to return any unused and empty sachets, and the number of unused sachets returned was counted and recorded on days 11–14. A measure of patch adherence was calculated as the number of sachets dispensed minus the number returned, divided by 10 (i.e., the total number of prescribed doses). If a patient dropped out of the treatment and failed to return dispensed sachets, the sachets were assumed not to have been used and were treated in the same way as returns. For the patient who dropped out of treatment in the first postrandomization of the study and never returned any sachets, this variable was coded as 0% adherence. This method of data collection for the measurement of sachet adherence is similar to pill count [21].

### 2.7. Overall Therapeutic Cure

Overall, the therapeutic cure was defined as meeting the criteria for both clinical and microbiological cure/investigational cure after treatment on days 11–14 from baseline. The microbiological test included vaginal saline wet mount test, pH determination, pus cell count, and whiff test with 10% KOH to confirm bacterial vaginosis with Amsel criteria, trichomoniasis, candidiasis, and uPID.

Clinical cure was defined as a patient who had normal vaginal discharge, negative KOH test, normal p^H^, and no uterine/cervical motion. Microbiological/investigational cure were defined as a normal vaginal cytology on vaginal smear and normal Pap smear report.

### 2.8. Randomization, Allocation, and Masking

A simple random sampling was used to randomly assign the patients into two groups. The allocation sequence was generated by random allocation software (RAS) with a single block with an allocation ratio of 1 : 1. An open list of random numbers was used through the order of randomization and until the interventions were assigned to the patient, it was concealed from the first researcher. The matching and masking were done by supplying the medicine in the same aluminium sachets.

### 2.9. Sample Size

The sample size of a total of 67 participants (*n*1 = 33, *n*2 = 34) was required that was calculated based on the proportion of cure 34% and 50% obtained from the previous study [22]. Hence, the sample size was taken as 66 including 10% dropout.

### 2.10. Statistical Analysis

For analysis of the data, the statistical software SPSS 22.0 ver 3.2.2 were used. Mean ± SD was used for results from continuous measurements and number (%) for categorical measurements. For all statistical tests, the test of significance was 5%, 95% confidence interval, and 80% power of the study, a two-sided *p* value. The Chi-square test or Fisher's tests were utilized for comparison of the proportions. For intragroup comparisons, a paired Student's *t*-test or Wilcoxon matched paired test depends on the skewness of data. The intergroup comparison using Student's *t*-test and Mann–Whitney *U* test for normally distributed data and skewed data, respectively, was performed. The ITT principle was performed for all efficacy variables using data from all randomized subjects with at least one postrandomization outcome measure. The last observation carried forward method was used to impute the missing data.

## 3. Results

### 3.1. Recruitment and Follow-Up

**Figure 2:**
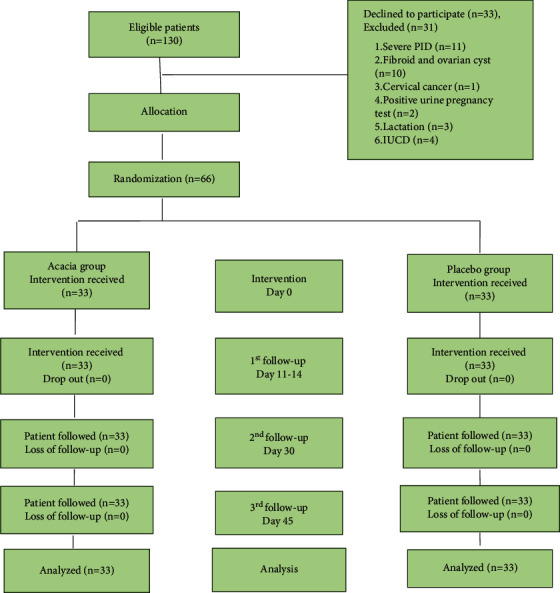
Flow chart of patients through the study according to consort statement.

The recruitment of patients was initiated on 1 March 2018 and completed on 8 November 2018. Initially, a total of 130 patients were screened, of which 64 were excluded (31 were ineligible, and 33 patients refused to participate). Thus, 33 patients were randomly allocated to each group. The flowchart for the enrolment of patients is shown in Figure 2.

### 3.2. Participant

The majority of the patients (96.96%) in each group were from an urban area. The mean age was 28.66 ± 5.76 and 30.57 ± 5.47 years in the *Acacia* and placebo group. The socio-demographic and reproductive characteristics are summarized in Table 1.

### 3.3. Primary Outcomes

#### 3.3.1. VAS Score of Abnormal Vaginal Discharge (AVD) and Its Associated Symptoms

*Acacia* group showed improvement in the mean VAS score for AVD and its associated symptoms after treatment. The intergroup comparison at each follow-up was statistically significant, *p* < 0.0001 (Table 2).

#### 3.3.2. Modified McCormack Pain Scale for Abdominal Pain and Rebound Tenderness

The mean score for the Modified McCormack Pain Scale (McPS) for abdominal pain and rebound tenderness on Day 11 was 1.06 ± 1.36 in the acacia and 1.96 ± 1.28 in the placebo group (*P*=0.01, statistically significant) (Table 2).

### 3.4. Secondary Outcome Measures

#### 3.4.1. EQ-5D-5L Health Questionnaire for HRQoL

**Figure 3:**
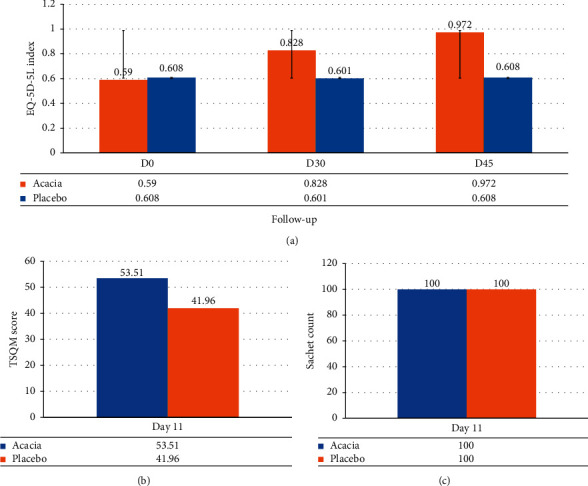
Depiction of secondary outcomes: (a) EQ-5D-5 L score, (b) TSQM score, and (c) Sachet count.

At baseline, index level in the *Acacia* and placebo group was statistically insignificant (*p*=0281). The *Acacia* group showed statistically significant improvement than the placebo group in EQ-5D-5L (*p* < 0.001). The *Acacia* group showed a statistically significant difference at posttreatment compared to baseline (*p* < 0.001), whereas it was insignificant in the placebo group (*p* > 0.05). The tests used were Mann–Whitney U test and Wilcoxon matched paired test for intergroup and intragroup comparison, respectively (Figure 3).

#### 3.4.2. Treatment Satisfaction Questionnaire for Medication (TSQM)

On Day 11, the mean score of TSQM was 53.51 ± 4.34 and 41.96 ± 3.64 in the *Acacia* and placebo groups, respectively, statistically extremely significant (*p* < 0.001) in the *Acacia* group (Figure 3).

#### 3.4.3. Sachet's Count for Compliance

The compliance in both groups was 100%

#### 3.4.4. Microbiological Test

The microbiological test on Day 11 was statistically significant (*p* < 0.0001) when compared to Day 0 in the *Acacia* group; however, in the placebo group, it was insignificant (*p* > 0.05) (Table 3).

#### 3.4.5. Overall Therapeutic Outcome

In the *Acacia* group, the therapeutic cure for bacterial vaginosis, cervicitis, and PID was 100% (*n* = 7/7), 45.45% (*n* = 10/22), and 71.42% (*n* = 5/7), respectively, whereas in the control group none of the patients had responded to the treatment (Table 4).

Data presented were as follows: no (%) or mean ± SD; ^a^*p* < 0.0001 considered extremely significant on Day 11 from Day 0 in the *Acacia* group; ^b^*p* > 0.05 considered not significant on Day 11 from Day 0 in the placebo group; tests used were as follows: Fisher's exact test and Wilcoxon matched paired test.

## 4. Discussion

### 4.1. Major Findings

This study is the first of its kind as none of the studies until date as per the researcher's knowledge has conducted a trial on sitz bath and vaginal pessary of *A. arabica* pod powder in abnormal vaginal discharge syndromic management, its associated symptoms, and improving women's health-related quality of life (HRQoL). The interpretation of the results supports that there was a significant reduction in abnormal vaginal discharge, its associated symptoms, and improvement in HRQoL in patients in the *Acacia* group. The present study showed a significant reduction in all symptoms in the *Acacia* group; similarly, Salhan et al. also reported a significant reduction in lower abdominal pain, dysuria, and vaginal itching with Praneem vaginal tablets [3]. In the *Acacia* group, the therapeutic cure for bacterial vaginosis, cervicitis, and PID was 100% (*n* = 7/7), 45.45% (*n* = 10/22), and 71.42% (*n* = 5/7), respectively, and none had trichomoniasis and a significant reduction in the symptoms. Likewise, Patel et al. showed that Ginlac-V pessary showed 100% efficacious in bacterial vaginosis and overall symptomatic relief was 82% in symptomatic vaginal discharge [17] and Motlagh et al. reported a 94% response with oral metronidazole plus *Prangos ferulacea* vaginal cream [19]. The present study showed a significant decrease in VAS score for low backache, vaginal discharge, and lower abdominal pain in the *Acacia* group; similarly, Bhat and Begum (2017) also reported a significant reduction in VAS score of symptoms for syndromic management of abnormal vaginal discharge with Unani formula [5].

### 4.2. Interpretation and Justification

Abnormal vaginal discharge is the commonest symptom for women in India to pursue care. *Acacia* pod extract was efficacious in the syndromic management of AVD, its associated symptoms, and improved HRQoL as it has anti-inflammatory (*Muhallil al-Waram*), astringent (*Qabiz*), and antiseptic (*Daf'-i-Taffun*) properties [6, 7]. Furthermore, *in vitro* and *in vivo* pharmacological studies have also proven anti-inflammatory [10, 11, 14], analgesic [10, 11], antimicrobial [23, 24], antispasmodic [25], diuretics [11], antioxidant properties [9, 13, 26], and antiseptic properties [10–14] of the pods. The aforementioned pharmacological properties are credited to the presence of phytoconstituents such as tannins, alkaloids, organic acids, flavonoids, polyphenolic compounds, volatile oils, glycosides, and coumarins [8, 9, 11, 13]. The methanolic pod extract of *A. arabica* showed significant inhibition against Gram-positive and Gram-negative species *in vitro* study [23]. Similarly, Satish et al. also reported antibacterial and antifungal activity of the methanolic extracts of *A. arabica* pods, and the highest activity was against *S. aureus*, *E. coli,* and *A. Niger* [8]. Another study also showed that the methanolic extract of pods had inhibitory activity against *P. aeruginosa*, *E. coli*, and *S. aureus* [24]. A study showed that the antimicrobial activity of the *Acacia* pods is probably due to polyphenolic compounds, and/or volatile oils cause inhibition of various microorganisms [8]. Phenol is established as a chemical antiseptic. The astringent effect of the pods is due to the presence of tannins [11]. Another study reported that the antioxidant and antibacterial activities are attributed to the presence of proteins and/or flavonoids and high total phenolic content [27]. The pods contain phytochemicals such as catechin, catechin 5-O-gallate, gallic acid, methyl gallate, 1-O-galloyl-*β*-D-glucose, gallocatechin 5-O-gallate, 1-6-di-O-galloyl-*β*-D-glucose, and digallic acid [28]. These phytochemicals are possibly accountable for the experiential activity. For example, tannins therapeutically have antiseptic properties and their precipitating activity is used in detecting alkaloids, proteins, and gelatin. Flavonoids and phenolic compounds are frequently found effective *in vitro* as antimicrobial substances against various microorganisms. They are plant metabolites with at least one hydroxyl group. Gallic acid by the mechanism of action in *E. coli, S. aureus, P. aeruginosa, and Listeria monocytogenes* led to “permanent changes in membrane properties through the decrease of negative surface charge, hydrophobicity changes, and pore formation in the cell membranes or local rupture with resulting leakage of essential intracellular constituents.” Oladous et al. (2019) reported that although gallic acid and methyl gallate have better activity than the crude extract, catechin was the most active compound against *S. aureus, E. coli, P. aeruginosa,* and clinical isolates of *K. pneumonia, Candida albicans, S. typhi,* and *B. subtilis* organisms [29].

The infective vaginal discharge not only affects women's routine physical and social activities but also their mental health and all aspects of a woman's life, thereby affecting HRQoL negatively. The positive effect of *A. arabica* pods on mood, headache, fatigue, and energy level is due to its antioxidant properties [9, 13, 26]. Furthermore, to explain that the reduction of associated symptoms may be attributed to its reported scientifically proven pharmacological activities such as astringent, anti-inflammatory, analgesic, and diuretic properties [8–14]. The *Acacia* group did not show any adverse effects during and after the completion of this trial.

### 4.3. Strengths of the Study

This is the first of its kind single-blind, randomized, placebo-controlled study using traditional regimen methods, sitz bath, and vaginal pessary of *Acacia* pods were efficacious for abnormal vaginal discharge syndromic management, its associated symptoms, and women's HRQoL. There were good patient's retention and compliance with the protocol. Overall therapeutic cure for the disease was also seen.

### 4.4. Limitations and Future Recommendations

Because of time constraints and lack of infrastructure, laboratory tests such as vaginal and cervical swab culture, nucleic acid amplification test (NAAT), and endometrial biopsy to verify the efficacy of the results were not possible. Furthermore, double-blind, phase IV clinical trials with larger samples are recommended.

## 5. Conclusion

The result of this study indicates that an *Acacia* pod's sitz bath and vaginal pessary were effective for syndromic management of abnormal vaginal discharge, its associated symptoms, and improving women's HRQoL. Furthermore, it is safe, well-accepted, and tolerated by the patients.

## Figures and Tables

**Table 1:** Baseline and socio-economic characteristics.

Variables	*Acacia* group (*n* = 33)	Placebo group (*n* = 33)	*p* value
Age (year)	28.66 ± 5.76	30.57 ± 5.47	0.17^c^
Urban	32 (96.96)	32(96.96)	1.00^a^
Past menstrual cycle			0.282^a^
Irregular	6(18.18)	3(9.09)	
Regular	27(81.81)	30(90.90)	
Duration of abnormal Vaginal discharge (days)	15.51 ± 11.85	14.72 ± 13.74	0.65^b^
Socio-economic status			0.71^a^
Upper middle (II)	13 (39.39)	16(48.48)	
Lower middle (III)	13 (39.39)	12(36.36)	
Upper lower (IV)	7 (21.21)	5(15.15)	
Height(cm)	156.28 ± 5.40	154.22 ± 4.73	0.051^c^
Weight(Kg)	61.09 ± 13.42	26.2 ± 5.89	0.714^c^
BMI (kg/m2)	24.98 ± 5.19	26.2 ± 5.89	0.376^c^

Data presented were as follows: mean ± SD or no (%); *p* > 0.05, considered not significant; tests used were as follows: ^a^Fisher's exact test; ^b^Chi-squared test; ^c^Mann–Whitney *U* test.

**Table 2:** Primary Outcome: VAS scoring of symptoms and the Modified McCormack Pain Scale in the *Acacia* and the control group.

Primary outcome	*Acacia* group (*n* = 33)	Placebo group (*n* = 33)	*p* value
VAS for LAP and LBA			
Day 0	4.66 ± 1.68	5.18 ± 1.26	0.26
Days 11–14	1.36 ± 1.72	5.06 ± 1.27	<0.0001
Day 45	0.63 ± 1.02^a^	4.90 ± 1.33^b^	<0.0001
VAS for abnormal vaginal discharge			
Day 0	6.39 ± 0.70	6.12 ± 0.59	0.39
Days 11–14	1.51 ± 2.04	6 ± 0.61	<0.0001
Day 45	0.60 ± 1.19^a^	6 ± 0.61^b^	<0.0001
VAS for dyspareunia			
Day 0	1.54 ± 2.32	1.75 ± 2.34	0.83
Days 11–14	0.15 ± 0.44	1.72 ± 2.34	0.02
Day 45	0.03 ± 0.17^a^	1.60 ± 2.20^b^	0.007
VAS for dysuria			
Day 0	1.69 ± 2.43	1.84 ± 2.43	0.74
Days 11–14	1.69 ± 2.43	1.81 ± 2.39	0.04
Day 45	0.09 ± 0.29^a^	1.66 ± 2.21^b^	0.008
VAS for burning micturition			
Day 0	3.48 ± 2.48	3 ± 2.44	0.38
Days 11–14	0.75 ± 1.03	2.84 ± 2.34	0.0007
Day 45	0.33 ± 0.64^a^	2.69 ± 2.24^b^	0.0001
VAS for vulvar irritation			
Day 0	3.18 ± 2.55	3 ± 2.72	0.94
Days 11–14	0.87 ± 1.34	2.87 ± 2.64	0.003
Day 45	0.18 ± 0.46^a^	2.84 ± 2.60^b^	<0.0001
VAS for vulvar itching			
Day 0	4.63 ± 2.14	3.90 ± 2.45	0.18
Days 11–14	1.42 ± 1.73	3.78 ± 2.23	<0.0001
Day 45	0.39 ± 0.65^a^	3.63 ± 2.35^b^	<0.0001
Modified McCormack Pain Scale (McPS) for abdominal tenderness			
Day 0	2.36 ± 1.43	2.36 ± 1.43	0.43
Days 11–14	1.06 ± 1.36	1.96 ± 1.28	0.01
Day 45	0.33 ± 0.76^a^	1.93 ± 1.24^b^	<0.0001

Data presented were as follows: mean ± SD; ^a^*p* < 0.0001 considered extremely significant on Day 11 and Day 30 from Day 0 in the *Acacia* group. ^b^*p* > 0.05 considered not significant on days 11–14 and Day 45 from Day 0 in the placebo group; tests used were as follows: Wilcoxon matched paired test VAS : visual analogue scale; LAP : lower abdominal pain; LBA : low backache.

**Table 3:** Microbiological evaluation in the *Acacia* and placebo groups.

Investigation	*Acacia* group (*n* = 33)		*p* value	Placebo group (*n* = 33)		*p* value
	Day 0	Day 11–14		Day 0	Day 11-14	
Amsel's criteria						
Whiff test			0.02			1.00
Negative	24 (72.72)	31 (93.93)		25 (75.75)	25 (75.75)	
Positive	9 (27.27)	2 (6.06)		8 (24.24)	8 (24.24)	
Clue cells			0.02			1
Negative	24 (72.72)	31 (93.93)		24 (72.72)	24(72.72)	
Positive	9 (27.27)	2 (6.06)		9 (27.27)	9 (27.27)	
Vaginal pH	4.54 ± 0.46	3.93 ± 0.48^a^	<0.0001	4.57 ± 0.47	4.59 ± 0.44^b^	0.78
Vaginal wet mount test for microscopic investigation of vaginal discharge						
KOH slide for hyphae			1.00			1.00
Present	0 (0)	0 (0)		1 (3.03)	1 (3.03)	
Absent	33 (100)	33 (100)		32 (96.96)	32 (96.96)	
Normal saline test for trichomonas			1.00			1.00
Absent	33 (100)	33 (100)		33 (100)	33( 100)	
Present	0	0		0	0	
Pus cells (hpf)			<0.001			0.93
<10	3 (9.09)	24 (72.72)		5 (15.15)	5 (15.15)	
11–20	6 (18.18)	6 (18.18)		8 (24.24)	7 (21.21)	
21–30	21 (63.63)	2 (6.06)		15 (45.45)	14 (42.42)	
>30	3 (9.09)	1 (3.03)		5 (15.15)	7 (21.21)	
Pus cells (hpf)	22.96 ± 8.48	8.21 ± 7.85	<0.001	23.15 ± 7.32	22.15 ± 8.7	0.47
Pap smear						
Normal	4 (12.12)	20 (60.60)	<0.001	4 (12.12)	8 (24.24)	0.63
Inflammatory	22 (66.66)	11 (33.33)		22 (66.66)	18 (54.54)	
BV	7 (21.21)	2 (6.06)		6 (18.18)	6 (18.18)	
Candidiasis	0(0)	0(0)^a^		1 (3.03)	1(3.03)^b^	

Data presented were as follows: no (%) or mean ± SD; ^a^*P* < 0.0001 considered extremely significant on day 11 from day 0 in the Acacia group; ^b^*P* > 0.05 considered not significant on day 11 from day 0 in the Placebo group; Tests used were as follows: fisher's exact test and Wilcoxon matched paired test.

**Table 4:** Overall therapeutic outcome (clinical cure, microbiological cure, and therapeutic cure in both groups).

Variables		Bacterial vaginosis		Candidiasis		Cervicitis		uPID	
Groups		AG	PG	AG	PG	AG	PG	AG	PG
No. of Pt		7(100)	6(100)	0	1(100)	22(100)	22(100)	7(100)	14(100)
Clinical cure									
Colour of discharge									
D0	White	7(100)	4(66.6)	0	1(100)	4(66.6)	6(27.27)	2(28.57)	4(28.57)
	Greyish	0(0)	0			15(68.18)	14(63.63)	2(28.57)	6(42.85)
	Yellowish	0(0)	2(33.33)			3(13.63)	2(9.09)	3(42.85)	4(28.57)
	Greenish	0(0)	0(0)			0(0)	0(0)	0(0)	0(0)
D11-14	Responded	7(100)	0	0	0	17(77.27)	0	5(71.42)	0
Odour of discharge									
D0	No-smell	0(0)	0(0)	—	0(0)	0(0)	0(0)	0(0)	0(0)
	Foul-smell	7(100)	0(0)	—	1(100)	19(86.36)	21(95.45)	5(71.42)	8(57.14)
	Fishy	0(0)	6(100)			3(13.63)	1(4.54)	2(28.57)	6(42.85)
D11-14	Responded	7(100)	0	0	0	20(90.90)	0	7(100)	0
Amount of discharge									
D0	Present	7(100)	6(100)	—	1(100)	22(100)	22(100)	7(100)	14(100)
D11-14	Responded	7(100)	0	—	0	10(45.45)	0	5(71.42)	0
D0	Ut. motion tenderness	—	—	—	—	17(77.27)	18(81.81)	7(100)	14(100)
D11-14	Responded	—	—	—	—	10(58.82)	0	5(71.42)	0
D0	McPS	—	—	—	—	—	—	7(100)	14(100)
D11	Responded	—	—	—	—	—	—	5(71.42)	0
Microbiological and investigational cure (vaginal smear, pH, pap smear)									
D0	pH > 4.5	6(85.7)	5(83.33)	—	—	—	—	—	—
D11-14	Responded	6(100)	0						
D0	Clue cells	7(100)	6(100)						
	Hyphae	—	—	—	1(100)	—	—	—	—
	Pus cells >10					22(100)	21 (95.45)	6(85.71)	13(92.8)
D11-14	Responded	7(100)	0		0	13(59.09)	0	6	7(100)
D0	Whiff test	7(100)	6(100)	—	—	—	—	—	7(100)
D11-14	Responded	7(100)	0	—	—	—	—	—	7(100)
D0	Pap smear	7(100)	6(100)	-	1(100)	22(100)	22(100)		7(100)
D11-14	Responded	7(100)	0	—	—	10(45.45)	0		7(100)
Therapeutic cure		7(100)	0	0	0	10(45.45)	0	5(71.42)	0

Data presented were as follows: no (%); AG : *Acacia* group; PG : placebo group; McPS : Modified McCormack Pain Scale; Pt : patient; uPID (uncomplicated pelvic inflammatory disease)

## Data Availability

The research is approved by the Institutional Ethics Committee (same has been added in the manuscript). The findings of this study are available from the corresponding author upon request.
